# Horner syndrome immediately after deep dissection of upper thyroid pole: a case report and review of the literature

**DOI:** 10.1515/iss-2023-0056

**Published:** 2024-02-02

**Authors:** Hongdan Chen, Yiceng Sun, Mi Tang, Fan Zhang

**Affiliations:** Department of Breast and Thyroid Surgery, Chongqing General Hospital, Chongqing University, Chongqing, China

**Keywords:** Horner syndrome, upper thyroid pole, endoscopic thyroidectomy, deep dissection, case report

## Abstract

**Objectives:**

Horner syndrome (HS) is a rare complication of thyroid surgery. However, the relationship between the occurrence of HS and thyroid upper pole injury is still not completely clear, and there are only few reports.

**Case presentation:**

A 24-year-old female underwent endoscopic thyroidectomy for thyroid papillary carcinoma. The intraoperative examination found that the upper pole of the thyroid was bleeding. During hemostasis, the ultrasonic knife consciously peeled too deep and stopped. The patient developed HS immediately after operation. We analyzed the association between deep dissection of the upper thyroid pole and an increase in the HS incidence rate through literature searches and anatomical relationships.

**Conclusions:**

Our case report discussed the potential relationship between the degree of thyroid upper pole dissection and the occurrence of HS in routine thyroid surgery, and provided a warning for the degree of thyroid upper pole dissection in the clinic to avoid HS.

## Background

Horner syndrome (HS) is a syndrome caused by cervical sympathetic nerve injury. It was officially named by Swiss ophthalmologists in 1869 [1]. Its main clinical manifestations are the classic triad of ptosis, pupil narrowing and facial anhidrosis [2], [3], [4]. The occurrence of HS is caused by many reasons, including carotid dissection, malignant tumors, severe trauma, etc [5], [6], [7], [8].

HS is a rare complication after thyroid surgery [9], and its incidence rate after thyroid cancer surgery is approximately 0.2 % [10]. Generally, the cervical sympathetic trunk runs behind the carotid sheath and contains 3–4 ganglia, including the superior cervical, middle cervical, inferior cervical/cervical thoracic and vertebral ganglia. The superior cervical ganglion is the largest ganglion in the cervical sympathetic trunk. It is considered that the superior cervical ganglion is located at the level of C1–C4 and behind the carotid sheath [11], [12], [13]. In thyroid surgery, the close and highly variable anatomical relationship between the thyroid and the cervical sympathetic nerve increases the risk of sympathetic nerve damage during thyroidectomy. However, the potential relationship between HS and a deep dissection of the upper pole of the thyroid has not been analyzed and reported, which means that this report may be used as a clinical reference to decrease the incidence rate of HS. In this study, we aimed to analyze the causes of HS immediately after a 24-year-old woman had deep upper thyroid pole dissection, we combined this information with the literature reports, and we put forward the relationship between these sources of information to provide a reference for the degree of upper thyroid pole dissection to reduce the complication of HS in the clinic.

## Case presentation

**Figure 1:**
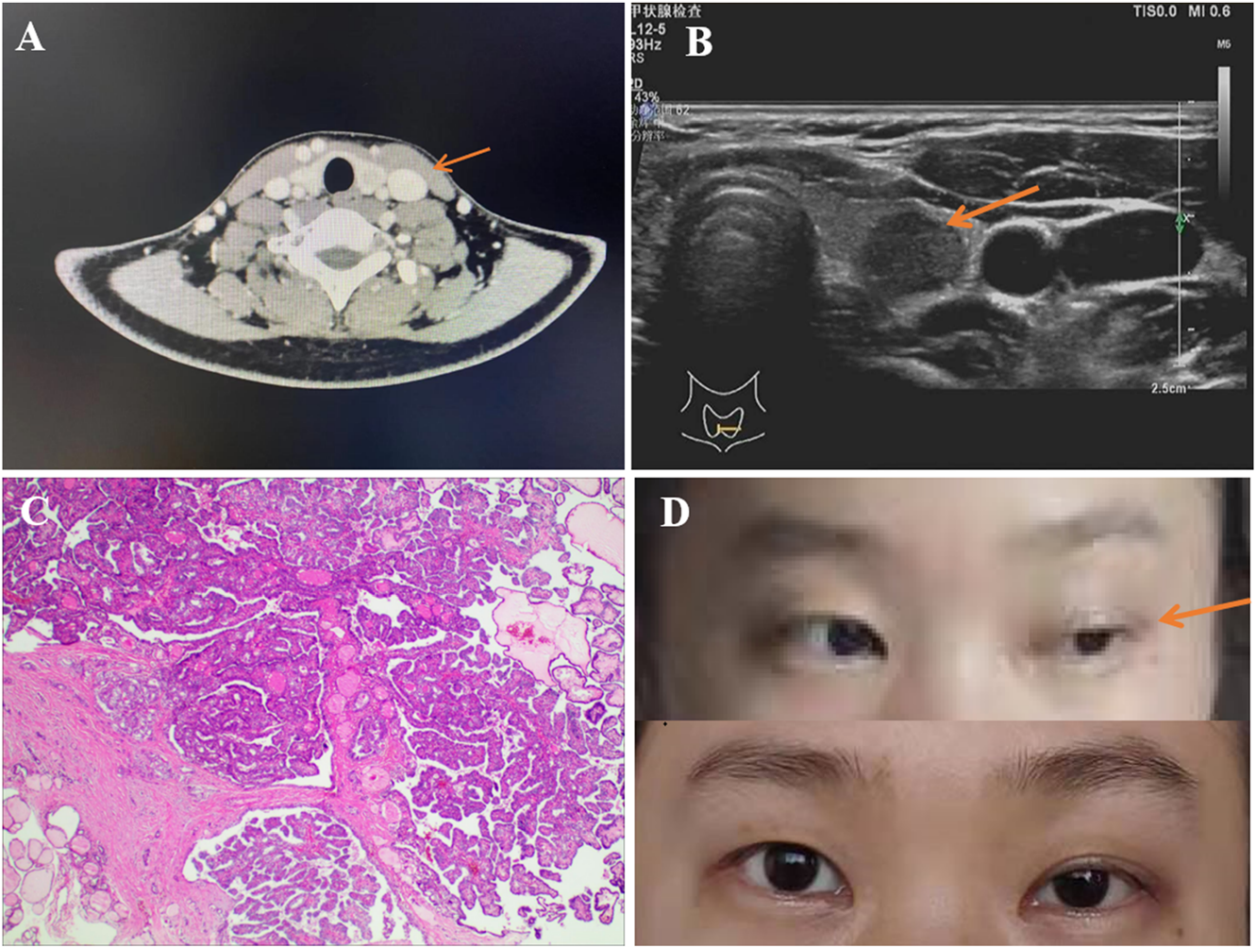
Correlative conditions of patients. (A) Computed tomography indicated a low-density shadow located at the lower part of the left thyroid, about 0.9*0.9*0.8 cm, weakly enhanced after enhancement; (B) ultrasound imaging demonstrated a nodule (0.96*0.86*0.1 cm) that was located at the lower part of the left thyroid gland; (C) microscopic image of papillary thyroid microcarcinoma (PTMC) from this patient, HE staining, ×4 magnification; (D) patient with left-sided Horner syndrome manifesting on postoperative 2 h with eyelid ptosis and anhidrosis.

A 24-year-old female patient with cough and sputum production underwent a chest CT examination, and a thyroid nodule was found (Figure 1A). After her cough was cured, the patient was admitted to the hospital for treatment of her thyroid gland. During the admission period, the patient did not report any relevant discomfort such as hoarseness, dyspnea or cough, and obvious abnormalities of physical examination were not found. A thyroid ultrasound showed the bilateral thyroid sizes were basically normal, and the left side was measured 4.70*1.20*1.60 cm. The nodule located at the left lobe of the inferior thyroid showed very low echo, measured 0.96*0.86 cm*1.0 cm, with a high possibility of malignancy (TI-RADS 4a) (Figure 1B). Fine needle biopsy suggested a thyroid tumor, the patient chose endoscopic thyroidectomy via total areola. So, the left lobe, isthmus and pyramidal lobe of the thyroid were resected. And considering this patient did not have extrathyroidal extension or ipsilateral node metastasis, we opted for ipsilateral central lymph node dissection.

**Figure 2:**
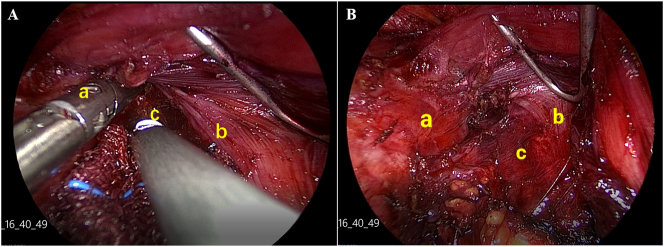
Stripping of upper thyroid pole during hemostasis in surgery. (A) Hemostasis of upper pole of thyroid; (B) after hemostasis of upper pole of thyroid ((a) scapula hyoid muscle, (b) cricothyroid muscle, (c) common carotid artery).

We performed thyroid surgery according to the routine procedure. The results of the pathology showed papillary thyroid carcinoma (Figure 1C). After removing the thyroid gland lobe, the branch of the superior thyroid artery started bleeding. The ultrasonic knife was used close the clamp branch of the common carotid artery to stop the bleeding (Figure 2). After hemostasis was obtained, the ultrasonic knife head was used below the level of the common carotid artery. However, the patient showed ptosis and anhidrosis 2 h after operation and was diagnosed with HS (Figure 1D). After 42 days of follow-up, the symptoms of HS were significantly improved without any measures. However, based on the standardized operation of the whole operation, the only difference between this case and other cases without immediate HS was that the upper pole dissection was too deep. At the same time, when the patient underwent central lymph node dissection, the cleaning range does not surpass the lower thyroid arteries, and the lower thyroid arteries are completely retained. The ultrasound also showed there was no obvious hematoma or swelling in the surgery area after surgery. The patient developed HS immediately after the operation, so we have to speculate about the relationship between the two.

## Discussion

The relationship between upper thyroid pole injury and HS is still unclear. This case suggested that a superior thyroid pole injury may be a potential cause of HS. To a certain extent, we found that deep dissection of the upper thyroid pole is likely to increase the incidence rate of HS, providing a reference for clinical practice. Interestingly, in a magnetic resonance imaging (3T-MRI) study of the superior cervical ganglion, 73 % of the superior cervical ganglion was confirmed at the C2–C4 level, located inside the internal carotid artery, and 27 % of the cases were located outside or behind the internal carotid artery [14]. At present, the incidence of the presence of middle cervical ganglion has not been unified. Civelek et al. observed the middle cervical ganglion in 74 % of cases, and it was mainly distributed in C5–C7 [11]. However, other studies have shown that the savings of the middle cervical nerve are 28.1–53.2 %, usually at the C3–C7 level, behind the carotid sheath and in front of the long neck muscle [15], [16], [17]. Therefore, we have reasons to believe that the sympathetic trunk may gradually travel from the back of the carotid sheath to the inside of the internal carotid artery at the level of the middle cervical ganglion. When dealing with the upper thyroid pole, especially during endoscopic surgery, due to the limited operation space, it is necessary to pull on the carotid sheath to expose the visual field. When the retractor is used to open the carotid sheath, it is very likely to expose the superior cervical ganglion and sympathetic trunk inside the internal carotid artery. Moreover, prolonged retractor traction and compression and the use of energy instruments may damage the exposed sympathetic nerve.

The sympathetic nerve is very sensitive to heat exposure. Endoscopic thyroid surgery depends more on energy instruments than open surgery does. Frequent use of energy instruments undoubtedly increases the probability of sympathetic nerve thermal energy injury, and the degree of injury depends on the heating time. Hallgrimsson and others believed that the distance between the energy instrument and the important tissues should be more than 5 mm, which is a relatively safe distance that can effectively reduce the exposure and damage of thermal energy [18]. Considering the thermal radiation of the ultrasound knife, perhaps we could choose to use suture or biological clamp hemostasis to avoid nerve damage. The thyroid branch may have communicating branches with the recurrent laryngeal nerve in some patients. When the recurrent laryngeal nerve is dissected, improper surgical operations may damage the sympathetic nerve [19]. When dissecting the recurrent laryngeal nerve, an improper operation may damage the sympathetic nerve. The middle cervical ganglion and its thyroid branches are closely related to the inferior thyroid artery. The inferior thyroid artery can pass through the middle cervical ganglion forward or backward. This close relationship makes it very easy to damage the middle cervical ganglion when ligating the inferior thyroid artery [20]. In addition, it has been reported that the inferior thyroid artery or its branches supply the carotid sympathetic nerve chain, and ligation of this artery may cause ischemia [21, 22].

In our preliminary research, we found that endoscopic surgery has a higher incidence of HS [23]. We summarized the following reasons: first, endoscopic surgery space is narrower, especially when it is treated with the upper pole of the thyroid, exposure is more difficult. It is easy to cross the carotid sheath during operation, and some mutant superior cervical ganglion is lighter and more easily damaged here. Once the upper pole is peeled too deeply and exceeds the level of the carotid sheath, HS is prone to appear after the operation due to the exposure and injury related to the superior cervical ganglion. When dealing with the middle and lower poles of the thyroid gland, the surgical space is greater, and it is easier to protect the sympathetic nerves. Secondly, endoscopic hooks are sharper, and they may cause hematoma compression nerve when pulling the cervical sheath. Finally, endoscopic surgery has a longer learning curve, and the unfamiliarity of early surgery is more likely to cause damage. In our case, HS recovery time is long, considering that thermal damage may be greater, it was suggested that the dissection in the upper pole of the thyroid gland was closely related to the cervical sympathetic nerve injury, which provides new ideas for the protection of sympathetic nerves.

## Conclusions

The dissection in the upper pole of the thyroid gland was closely related to the cervical sympathetic nerve injury, which provides new ideas for the protection of sympathetic nerves. Once the upper pole is peeled too deeply and exceeds the level of the carotid sheath, HS is prone to appear after the operation due to the exposure and injury related to the superior cervical ganglion. The protection of the sympathetic nerve requires the correct identification of both the course of the nerve and the anatomical plane. During the operation, excessive pulling of the carotid sheath should be avoided, and at the same time, a dissection that is too deep should be avoided and we should strict close dissection to the thyroid in benign cases in malignant cases as close as possible in oncological intention.

## References

[1] Amonoo-Kuofi HS (1999). Horner’s syndrome revisited: with an update of the central pathway. Clin Anat.

[2] Fustes OJH, Kay CSK, Lorenzoni PJ, Ducci RDP, Werneck LC, Scola RH (2021). Horner syndrome: tribute to Professor Horner on his 190th birthday. Arq Neuropsiquiatr.

[3] Koka K, Patel BC (2021). Ptosis correction. StatPearls.

[4] Khan Z, Bollu PC (2021). Horner syndrome. StatPearls.

[5] Hebant B, Lefaucheur R, Delpierre C, Ozlem OW (2021). Horner syndrome as the sole manifestation of an ipsilateral intrapetrous internal carotid artery dissection. Rev Neurol.

[6] Mansukhani SA, Eckel LJ, Wu KY, Hassan MB, Van Loon JA, Chen JJ (2021). Horner syndrome due to internal carotid artery dissection with normal vascular imaging: a radiological conundrum. J Neuro Ophthalmol.

[7] Azharudeen M, Selvaraj J, Pillai V, Meyyappan J, Veeranki V (2021). Malignant peripheral nerve sheath tumor presenting as Horner’s syndrome. Cureus.

[8] Ozel SK, Kazez A (2005). Horner syndrome due to first rib fracture after major thoracic trauma. J Pediatr Surg.

[9] Demiral M, Binay C, Simsek E, Ilhan H (2017). Horner syndrome secondary to thyroid surgery. Case Rep Endocrinol.

[10] Lee YS, Nam KH, Chung WY, Chang HS, Park CS (2010). Postoperative complications of thyroid cancer in a single center experience. J Korean Med Sci.

[11] Civelek E, Karasu A, Cansever T, Hepgul K, Kiris T, Sabancı A (2008). Surgical anatomy of the cervical sympathetic trunk during anterolateral approach to cervical spine. Eur Spine J.

[12] Tubbs RS, Salter EG, Oakes WJ (2005). Anatomic landmarks for nerves of the neck: a vade mecum for neurosurgeons. Neurosurgery.

[13] Yin Z, Yin J, Cai J, Tao S, Xiaojian C (2015). Neuroanatomy and clinical analysis of the cervical sympathetic trunk and longus colli. J Biomed Res.

[14] Lee JY, Lee JH, Song JS, Song MJ, Hwang SJ, Yoon RG (2016). Superior cervical sympathetic ganglion: normal imaging appearance on 3T-MRI. Korean J Radiol.

[15] Kiray A, Arman C, Naderi S, Güvencer M, Korman E (2005). Surgical anatomy of the cervical sympathetic trunk. Clin Anat.

[16] Shin JE, Baek JH, Ha EJ, Choi YJ, Choi WJ, Lee JH (2015). Ultrasound features of middle cervical sympathetic ganglion. Clin J Pain.

[17] Kawashima T (2005). The autonomic nervous system of the human heart with special reference to its origin, course, and peripheral distribution. Anat Embryol.

[18] Hallgrimsson P, Lovén L, Westerdahl J, Bergenfelz A (2008). Use of the harmonic scalpel versus conventional haemostatic techniques in patients with Grave disease undergoing total thyroidectomy: a prospective randomised controlled trial. Langenbeck’s Arch Surg.

[19] Reeve TS, Coupland GA, Johnson DC, Buddee FW (1969). The recurrent and external laryngeal nerves in thyroidectomy. Med J Aust.

[20] Seneviratne SA, Kumara DS, Drahaman AM (2016). Horner’s syndrome: an unusual complication of thyroidectomy: a case report. J Med Case Rep.

[21] Meng K, Tian W, Lv Z, Song X (2015). Horner’s syndrome subsequent to minimally invasive video-assisted thyroidectomy in two patients. Oncol Lett.

[22] Solomon P, Irish J, Gullane P (1993). Horner’s syndrome following a thyroidectomy. J Otolaryngol.

[23] Tang M, Yin S, Yang Z, Sun Y, Chen H, Zhang F (2022). Horner syndrome after thyroid-related surgery: a review. Langenbeck’s Arch Surg.

